# Correlation of Oncotype DX Recurrence Score with Histomorphology and Immunohistochemistry in over 500 Patients

**DOI:** 10.1155/2017/1257078

**Published:** 2017-01-12

**Authors:** Matthew G. Hanna, Ira J. Bleiweiss, Anupma Nayak, Shabnam Jaffer

**Affiliations:** ^1^Department of Pathology, University of Pittsburgh, School of Medicine, S-417 BST, 200 Lothrop Street, Pittsburgh, PA 15261, USA; ^2^Department of Pathology, Hospital of the University of Pennsylvania, University of Pennsylvania, Founders 6, 3400 Spruce St., Philadelphia, PA 19104, USA; ^3^Department of Pathology, Icahn School of Medicine at Mount Sinai, One Gustave L Levy Pl., P.O. Box 1194, New York, NY 10029, USA

## Abstract

Oncotype Dx is used to determine the recurrence risk (RR) in patients with estrogen receptor positive (ER+) and lymph node negative (LN−) breast cancer. The RR is divided into low (0–17), intermediate (18–30), and high (31) to predict chemotherapy benefit. Our goal was to determine the association between histomorphology, immunohistochemistry, and RR. We retrospectively identified 536 patients with ER+ and LN− breast cancers that underwent Oncotype testing from 2006 to 2013. Tumor size ranged from 0.2 cm to 6.5 cm (mean = 1.3 cm) and was uniform in all 3 categories. The carcinomas were as follows: ductal = 63.2%, lobular = 11.1%, and mixed = 35.7%. The RR correlated with the Nottingham grade. Increasing RR was inversely related to PR positivity but directly to Her2 positivity. Of the morphologic parameters, a tubular(lobular) morphology correlated only with low-intermediate scores and anaplastic type with intermediate-high scores. Other morphologies like micropapillary and mucinous were uniformly distributed in each category. Carcinomas with comedo intraductal carcinoma were more likely associated with high RR. Forty-four patients with either isolated tumor cells or micrometastases were evenly distributed amongst the 3 RR. While there was only 1 ER discrepancy between our immunohistochemistry (3+ 80%) and Oncotype, up to 8% of PR+ cases (mean = 15%, median = 5%) and 2% of HER2+ cases were undervalued by Oncotype.

## 1. Introduction

Oncotype Dx (Genomic Health, Redwood City, California) is a commercially available multigene reverse transcription polymerase chain reaction (RT-PCR) assay that is used to quantify the risk of distant recurrence and predict the benefits of chemotherapy in patients with stage I or II estrogen receptor positive (ER+), Her2 negative, and lymph node negative (LN−) invasive breast cancer treated with tamoxifen [1]. The results are reported in a numeric recurrence score (RS), from 0 to 100, corresponding to low (0–17), intermediate (18–30), and high scores (>30), with an average risk of distant recurrence of 6.8, 14.3, and 30.5%, respectively [1]. Its impact and indication are primarily in helping to identify which patients with ER+ LN− tumors need chemotherapy, since the majority of these patients will be disease free without adjuvant therapy. Despite the expense of the test, it is recommended by ASCO and NCCN in specific subsets of patients to offset the morbidity and costs related to unnecessary chemotherapy.

The Oncotype Dx gene panel consists of 21 genes: 16 cancer genes and 5 reference genes. The cancer genes are comprised of a proliferation set (5 genes: Ki67, STK15, Survivin, CCNB1, MYBL2), estrogen hormone receptor genes (5 genes: ER, PGR, BCL2, SCUBE2), HER2neu set (2 genes: GRB7, HER2), invasion set (2 genes: MMP11, CTSL2), and GSTM-1 (1 gene). An estimation of the Oncotype Dx RS can be achieved with routine histopathologic parameters including integrated mitotic activity and immunohistochemical protein expression of hormone receptors and HER2neu [2, 3]. This provides an alternate more economical and effective mechanism to assess the effects of most of the genes. Thus, we sought to amplify on this by attempting to identify any histomorphological and or immunohistochemical correlation with Oncotype Dx.

## 2. Methods

Using our pathology laboratory information system database, we retrospectively identified 536 patients with ER+ LN− breast cancers that underwent Oncotype DX testing at our institution from 2007 to 2013. Clinical information including age and tumor size was gathered in all cases. All cases were reviewed to evaluate Nottingham histologic grade (tubule formation, nuclear grade, and mitotic rate). Histomorphology was examined for any unusual variants, and immunohistochemical staining (ER, PR, and HER2neu) was checked on excision specimens. Hormone receptor scoring was performed using the percent of positive staining and intensity to calculate an Allred score; a score of 0–2 was considered negative, 3-4 weakly positive, and 5–8 strongly positive. Her2neu was scored as proposed by the most recent American Society of Clinical Pathology (ASCP) guidelines [4] as negative (0, 1+), equivocal (2+), and positive (3+, >10%). All equivocal tests were sent for FISH and deemed positive if gene amplification was >2.0. These values were compared with reports from Oncotype Dx, including RS obtained from Genomic Health reports.

## 3. Statistical Analysis 

Statistical analyses were performed using R version 3.0.3 (R Core Team (2013); R: A language and environment for statistical computing; R Foundation for Statistical Computing Vienna, Austria; ISBN 3-900051-07-0, URL http://www.R-project.org/). A statistically significant level of* p* value < 0.05 was defined. The Oncotype Dx data and data generated from the patient diagnosis reports from our institution were analyzed using chi-squared tests. Concordance statistics were also performed to identify any discrepancies between the results of both data sets. Each case was compared to identify discrepancies of ER, PR, and HER2neu hormone status. RS groups were stratified into three groups: low (0–17), intermediate (18–30), and high scores (>30). All histomorphological features of invasive, in situ, and benign disease were identified and compared to each recurrence risk group.

## 4. Results 

The age of the patients ranged from 33 to 77 (mean = 58 years). The Oncotype Dx RS were correlated by RS and other parameters as summarized in Table 1. RS was distributed as follows: low (0–17) = 299 (55.8%), intermediate (18–30) = 180 (33.6%), and high (≥31) = 57 (10.6%). The size of the tumors ranged from 0.2 cm to 6.5 cm (mean = 1.3 cm), and based on RS, average sizes were as follows: low = 1.4 cm, intermediate = 1.3 cm, and high = 1.2 cm. By subtype, the carcinomas could be divided as follows: ductal = 63.2%, lobular = 11.1%, and mixed = 25.7%. Using the Nottingham classification, the RS distributed across each recurrence risk group as follows: low RS: 3–5 = 19.7%, 6-7 = 67.3% 8-9 = 13%; intermediate: 3–5 = 8.3%, 6-7 = 68.9%, and 8-9 = 22.8%; and high: 6-7 = 34.3% and 8-9 = 66.7%.

Next, we studied the morphology of the carcinomas for the presence of any specific variant patterns and subclassified them by RS, respectively, as low, intermediate, and high as discussed below. Beginning with type, carcinomas were classified by RS as follows: invasive ductal: 187, 106, and 42; invasive lobular: 34, 21, and 5; mixed ductal and lobular: 80, 53, and 8. The invasive lobular components were further subdivided for the presence of variants and RS as follows: pleomorphic 17.4%, 21.7%, and 19.3% and histiocytoid 2.7%, 7.2%, and 1.8%. Other invasive carcinoma variant patterns included tubular and tubulolobular 16.4%, 8.9%, and 0%; micropapillary 14.7%, 16.7%, and 24.6%; mucinous 4.7%, 4.4%, and 3.5%; anaplastic 0%, 1.7%, and 7.0%. Associated DCIS, comedo type, was subdivided by score as follows: 3.3%, 6.1%, and 15.8%. Lymphatic invasion was subdivided by RS as follows: 24.1%, 18.9%, and 22.8%.

Although Oncotype Dx is primarily utilized in patients who are N0, due to physician requests, our series also included a small subset of stage I or II ER+ patients that had ITCs (25 patients = 4.7%) and micrometastases (19 patients = 3.6%). The age of these patients ranged from 34 to 81 years (mean = 57) and the size of the tumors ranged from 0.6 cm to 3.0 cm (mean = 1.4 cm). The RS were as follows: 17 (38.6%) cases (8 pN0i, 9 pN1mi) were categorized as low, 21 (47.8%) as intermediate (12 pN0i, 9 pN1mi), and 6 (13.6%) as high RS (5 pN0i, 1 pN1mi). By histologic grade, they were classified as follows: moderately differentiated (Nottingham grade 2) = 26 and poorly differentiated (Nottingham grade 3) = 18. Histopathologic parameters including size, presence of DCIS, lymphovascular invasion, and size of isolated tumor cells or micrometastases did not show any correlation with the RS.

Comparison of hormone analysis and HER2neu by immunohistochemistry (IHC) performed in our CAP approved laboratory and Oncotype Dx was done. Of note, the same paraffin block used for in house biomarker studies was used to cut additional blanks for Oncotype Dx. A corresponding h&e was also obtained for inspection prior to sending out the blanks. There was complete concordance of ER (99.8%) except for one case which was negative by Oncotype Dx but 3+ 80% by IHC. Progesterone receptor (PR) concordance rate was 91.4% and was distributed by RS as follows: low = 97.5%, intermediate = 76.0%, and high = 40.8%. There were 47 (8.7%) PR discrepant cases: 8 that were positive on Oncotype Dx and negative by IHC (all cases with 0% cells positive) and 38 that were negative by Oncotype Dx but positive by IHC. Of the latter, percentage positivity ranged from 1 to 95% (mean = 15%, median = 5%). The majority (91%) had an intensity of 1+, with 2 cases that were 2+ and 2 cases that were 3+. Seventy-three cases (13.6%) were PR negative by Oncotype Dx and IHC and their respective RS were subdivided as follows: low RS 7 (2.6%), intermediate RS 37 (29.9%), and high RS 29 (59.2%). Of the poorly differentiated tumors, PR negativity was seen to stratify by RS as follows: low = 1.34%, intermediate = 5%, and high = 38.6%.

Although Oncotype Dx is primarily utilized in patients who are Her2 negative, due to physician requests, our series also included a small subset of HER2 positive cases. Her2 was concordant in 98.3% cases and discrepant in 13 (2.7%) cases. Nine cases that were positive on IHC were either negative (5) or equivocal (4) on Oncotype Dx. Four cases that were negative on IHC were equivocal on Oncotype Dx. The Oncotype Dx positive cases were of predominantly high RS, with only 1.95% of intermediate score that was detected by IHC but not Oncotype Dx.

## 5. Discussion

It is well established that the Oncotype Dx RS can be used to quantify the risk of distant recurrence in patients with stages I and II ER+, Her2 negative, and LN negative invasive breast cancer who will be treated with tamoxifen [1]. Although RS helps decide which patients should be selected to receive adjuvant therapy, the test is associated with a high expense. As a result, various studies have attempted to search for alternative simpler and more economical ways of providing similar information. The use of routine pathologic and IHC features such as Nottingham classification and hormonal and HER2neu analysis has been repeatedly shown to have comparable value due to the genes involved in the calculation of the RS [2, 3]. In addition, others have incorporated Ki67 to better assess the proliferation rate over an H&E based mitotic rate [5, 6]. In fact, outcome based data from the TransATAC trial showed the efficacy of “IHC4” score using ER, PR, HER2neu, and Ki67 to predict outcome better than Oncotype Dx [7]. Studies have consistently shown that the combined use of PR and mitotic rate can serve as a surrogate marker for RS [8, 9]; this notion is further supported by our study. HER2neu positive cases, albeit less frequent due to a low incidence of triple positive cancers, were also associated with a high RS as seen in our series.

We studied the grade and morphology of each case in an attempt to identify any specific morphological variant of the carcinoma. Few studies with limited numbers have attempted to correlate morphology with RS [10–12], two out of three, limited only to invasive lobular carcinomas. Our series, comprised of 536 patients, is the largest and most diverse of its kind, comprised of invasive ductal and lobular carcinomas and their variants. We found that grade I carcinomas were exclusively associated with low to intermediate RS and grade 2 carcinomas predominated in the low to intermediate RS whereas grade 3 carcinomas had intermediate to high RS. Our analysis showed that variants of invasive ductal carcinoma such as micropapillary and mucinous carcinomas were uniformly distributed in each of the three RS. This is in contrast to the study by Bomeisl et al. [10] which showed micropapillary carcinomas having high RR and mucinous carcinomas with intermediate RS. We noticed that tubular and tubulolobular morphology correlated only with low and intermediate scores. Invasive lobular carcinomas showed a progression in the spectrum of RS, with more than half in the low end, with sequential decreases in the intermediate and high. This held true even when admixed with invasive ductal carcinoma. In contrast, the pleomorphic variant showed an inverse relationship with RS and predominated in the intermediate to high RS, suggesting an aggressive subtype. The anaplastic variants of invasive ductal carcinoma were exclusively associated with only intermediate and high scores. Lymphatic invasion did not correlate with score. Regarding DCIS associated with the invasive carcinomas, as might be expected, the comedo type was more likely associated with invasive carcinomas with high RS.

There was only 1 ER discrepancy between our IHC (3+ 80%) and Oncotype Dx; up to 8% of PR+ cases (mean % positivity = 50%, median = 20%) and 2% of HER2neu+ cases were undervalued by Oncotype Dx. Several studies have commented on the discrepancy rates between Oncotype Dx and IHC for ER, PR, and HER2neu status. Studies report 93–100% concordance with ER and 86–94.2% PR concordance rates [2, 3, 13, 14]. Oncotype Dx results most often undervalue ER and PR receptors when compared to IHC, as also seen in our study, making IHC evaluation by current interpretive guidelines more sensitive for detecting hormone receptor status. Even with relatively high (>90%) concordance rates between both modalities, studies show that some of the discordant cases may be due to contamination or interference by cellular stroma, inflammatory cells, or a biopsy cavity [5, 15]. Thus, patients could potentially not receive adjuvant endocrine chemotherapy if the Oncotype Dx results are interpreted in isolation by medical oncologists. Park et al. discuss clinical scenarios at their institution where the Oncotype Dx results were favored over the IHC, resulting in falsely omitting adjuvant therapy [6]. In addition, cases that were HER2neu negative or equivocal by Oncotype Dx were found to be Her2-FISH amplified, such that only one of the patients received trastuzumab. Dabbs et al. discussed five patients who also did not receive trastuzumab due to discordant results [16]. It is prudent to exercise caution when interpreting discrepant results since it may lead to inappropriate omission of antihormonal or anti-HER2neu therapy.

Studies have shown that PR negativity has an inverse relationship with Oncotype Dx RS, independent of Nottingham score [9, 17]. Our findings are similar to the study by Auerbach et al. who showed that a mitotic count greater than 1 combined with a negative PR result could serve as a marker for an intermediate or high Oncotype Dx RS [17]. Similarly, Allison et al. found an inverse relation between Nottingham grade combined with PR expression and RS [3]. In our series, there was discordance between PR and Oncotype Dx, particularly in poorly differentiated tumors. Univariate analysis has shown that PR negative compared with PR positive breast tumors have higher hazard ratios for relapse free survival and subsequently poorer prognosis [18]. In fact ER+ PR− breast tumors have increased expression of HER-1 and HER-2 and are more aggressive tumors than ER+ PR+ ones. The absence of PR in ER+ tumors may be a surrogate marker for increased growth factor signaling and may be a reason for ER+ PR− tumors having tamoxifen resistance [19]. In the ATAC trial, patients with ER+ PR− breast cancers given either Arimidex, tamoxifen, or a combination treatment saw a greater benefit in time to recurrence than the ER+ PR+ group [20, 21]. Several mechanisms can be attributed to the inverse relationship between PR and RS. PR expression implies an intact pathway for ER to perform its role as a nuclear transcription factor and thus be able to be targeted via ER modulators and downregulators. PR− cancers are characterized by increased growth factor signaling, activation of noncanonical ER signaling, and poorer outcome [22, 23]. As can be seen the RS is hormone dependent and suggests that ER+ PR− cancers maybe a distinct subtype of luminal breast cancers that may need more aggressive treatment.

It is well established that the Oncotype Dx RS helps guide the use of adjuvant therapy in patients with early stage ER+ LN− tumors, and this practice has been endorsed by both the National Comprehensive Cancer Network and American Society of Clinical Oncology [24]. On the other hand, patients with truly positive lymph nodes are generally offered both chemotherapy and adjuvant endocrine therapy. Thus, there has been little need to use a predictor such as Oncotype Dx to assess the need for chemotherapy in these patients with only few studies actually analyzing the role of Oncotype Dx in ER+ LN+ patients [25, 26]. Similar to earlier studies on ER+ LN− patients, few studies in this subgroup indicate that ER+ LN+ patients also had RS predictive of the benefit of anthracycline based chemotherapy, particularly in postmenopausal women. However, to date, there have been no formal assessments of the value of the RS in patients with LNs containing isolated tumor cells (ITCs) and micrometastases, although they were included in some series, albeit in small numbers, examining the efficacy of Oncotype Dx versus histology and IHC [3]. Our attempt to study this by isolating this group of patients from all of our patients tested with Oncotype Dx is the largest of its kind so far. Our results showed that patients with ITCs and micrometastases stratified evenly in all three RS but predominated (86%) in the low and intermediate scores. Using PR *H* score and mitotic grade, we found that the cases with PR *H* score > 250 and mitotic grade of 1 had low Oncotype Dx RS category and cases with PR *H*-score < 150 and mitotic grade of 3 were exclusive to high Oncotype Dx RS category. This indicates that Oncotype Dx may serve as a guide in predicting the utility of chemotherapy in patients with carcinomas that are ER+ with ITCs, since the biological potential of the latter is uncertain [27, 28].

A limitation of our study is its retrospective nature with no follow-up and inherent selection bias on the part of the ordering physicians, as not all carcinomas that were ER+ LN− underwent Oncotype Dx testing. Secondly, while we did not have a formula to calculate a RS, we were able to use certain histologic features to predict the behavior of a carcinoma. Using standard pathologic parameters, one can predict that grades 1 and 2 carcinomas were associated with low to intermediate RS whereas grade 3 carcinomas had intermediate to high RS. In addition, certain morphologies such as lobular and tubulolobular are most likely to be associated with low to intermediate RS, whereas other variants such as mucinous and micropapillary lacked such an association and in fact distributed evenly in each of the three RS. Additionally PR− carcinomas associated with high mitotic rates were more likely to be poorly differentiated and have high RS. Carcinomas with ITCs and micrometastases predominated in the low to intermediate scores. This illustrates that, within the family of varied breast cancer grades and morphologies, there may be heterogeneity even amongst the subsets as seen by the spectrum of RS within each subset. This emphasizes that morphology and biomarkers need to be used in association with the RS to determine the use of adjuvant therapy particularly in the intermediate RS. In summary, this is the first largest study of its kind showing morphologic and IHC correlation with Oncotype Dx and in particular its relevance in patients with ITCs or micrometastases. Further studies are needed to address the clinical relevance of testing in this patient population.

## Figures and Tables

**Table 1:** Summary of pathology findings correlated with recurrence score (RS).

	Low RS (0–17) *n* = 299	Intermediate RS (18–30) *n* = 150	High RS (≥31) *n* = 57	*p* value
SIZE	1.4 cm	1.3 cm	1.2 cm	0.997
Estrogen receptor+	100% (100%)	100% (100%)	99.8% (100%)	0.99
Progesterone receptor+	97.5% (97.5%)	76.0% (88.3%)	40.8% (61.2%)	<0.001
HER2Neu+^*∗*^	0% (0%)	0% (1.95%)	12.2% (26.5%)	<0.001
Nottingham (3–5)	19.7%	8.3%	0%	<0.001
Nottingham (6-7)	67.3%	68.9%	34.3%	<0.001
Nottingham (8-9)	13%	22.8%	66.7%	<0.001
Tubular ± lobular	16.4%	8.9%	0%	<0.001
Invasive lobular cancer, pleomorphic	17.4%	21.7%	19.3%	0.790
Invasive lobular cancer, histiocytoid	2.7%	7.2%	1.8%	0.110
Micropapillary	14.7%	16.7%	24.6%	0.233
Mucinous	4.7%	4.4%	3.5%	0.913
Anaplastic	0%	1.7%	7.0%	0.010
Lymphatic invasion	24.1%	18.9%	22.8%	0.716
Ductal carcinoma in situ, comedo type	3.3%	6.1%	15.8%	0.006

^*∗*^Oncotype DX score (in house score).
